# Data for life cycle assessment of legume biorefining for alcohol

**DOI:** 10.1016/j.dib.2019.104242

**Published:** 2019-08-08

**Authors:** Theophile Lienhardt, Kirsty Black, Sophie Saget, Marcela Porto Costa, David Chadwick, Robert Rees, Michael Williams, Charles Spillane, Pietro Iannetta, Graeme Walker, David Styles

**Affiliations:** a School of Environment, Natural Resources and Geography, Bangor University, Bangor LL57 2UW, UK, Wales; b Plant and AgriBiosciences Centre, Ryan Institute, National University Ireland Galway, Galway, Ireland; c Arbikie Distilling Ltd, Inverkeilor, Arbroath DD11 4UZ, Scotland, UK; d Division of Food & Drink, Abertay University, Dundee DD1 1HG, UK; e Ecological Sciences, The James Hutton Institute, Dundee DD2 5DA, Scotland, UK; f Yeast Research Group, Abertay University, Dundee, DD1 1HG, Scotland, UK; g Department of Botany, School of Natural Sciences, Trinity College Dublin, Dublin 2, Ireland; h Scotland's Rural College, West Mains Road, Edinburgh EH9 3JG, Scotland, UK

**Keywords:** Pea, Legumes, Life cycle assessment, LCA, Animal feed, DDGS, Distillation, Ethanol

## Abstract

Benchmarking the environmental sustainability of alcohol produced from legume starch against alcohol produced from cereal grains requires considering of crop production, nutrient cycling and use of protein-rich co-products via life cycle assessment. This article describes the mass balance flows behind the life cycle inventories for gin produced from wheat and peas (Pisum sativum L.) in an associated article summarising the environmental footprints of wheat- and pea-gin [1], and also presents detailed supplementary results. Activity data were collected from interviews with actors along the entire gin value chain including a distillery manager and ingredient and packaging suppliers. Important fertiliser and animal-feed substitution effects of co-product use were derived using detailed information and models on nutrient flows and animal feed composition, along with linear optimisation modelling. Secondary data on environmental burdens of specific materials and processes were obtained from the Ecoinvent v3.4 life cycle assessment database. This article provides a basis for further quantitative evaluation of the environmental sustainability of legume-alcohol value chains.

**Table:** Specifications Table

Subject	Environmental Science (General)
Specific subject area	Life cycle assessment of agri-food chains
Type of data	Text & Tables
How data were acquired	Mass flow and life cycle inventory data were collated from primary and secondary sources, including: (i) interviews with value chain stakeholders to identify quantities, origins and transport of inputs used in gin production; (ii) statistics on agronomic inputs and yields of wheat and pea crops; (iii) commercial LCA databases, primarily Ecoinvent v3.5.
Data format	Data presented are collated raw and processed data that have been converted into mass balance flows for wheat and pea-gin value chains, and analysed results.
Parameters for data collection	Mass flows of materials and constituent nutrients in value chains of wheat- and pea- gin production.
Description of data collection	Primary data were collated via face-to-face, telephone and email communication with stakeholders. Secondary data were collated via searches of the academic literature (Google Scholar) and through access to the commercial Ecoinvent v.3.5 database using Open LCA v1.7.
Data source location	Data collection related to gin production in the Arbikie Distillery, Inverkeilor, Arbroath, Scotland Latitude: 56.64662 Longitude: −2.55632
Data accessibility	With the article
Related research article	Theophile Lienhardt, Kirsty Black, Sophie Saget, Marcela Porto Costa, David Chadwick, Robert Rees, Mike Williams, Charles Spillane, Pietro Iannetta, Graeme Walker, David Styles Just the tonic! Legume biorefining for alcohol has the potential to reduce Europe's protein deficit and mitigate climate change Environment International DOI pending

**Table:** 

**Value of the Data** • These data provide detailed life cycle inventories and full life cycle assessment results for gin made from wheat and peas, including potential substitution of fertilisers and animal feed • Data are useful for any academics studying gin value chains, e.g. to calculate environmental footprints or economic profiles, and for any stakeholders interested in the environmental sustainability of gin and other alcohol value chains • Data may be used to parameterise basic grain- and legume-life cycle inventories as a basis for new (legume)-alcohol LCAs • These high resolution data provide insight into important processes underpinning the life cycle inventories summarised in Lienhardt et al. [1], and indicate the full range of life cycle assessment results derived from sensitivity analyses

## 1 Data

Primary and secondary data used to build the life cycle inventories for wheat- and pea-gin are described in the next section, with key information summarised in Table 1, Table 2, Table 3, Table 4, Table 5, Table 6, Table 7, Table 8.

**Table 1:** Main inputs to the distillation process for one batch of gin.

Input/output	Unit	Wheat gin	Pea gin
Wheat grain	kg	2703	
Pea grist	kg		2782
Water	L	25 454	
Yeast	kg	13.5	
A-amylase	kg	1.2	
Glucoamylase	kg	3.3	
Kerosene	L	870	
Electricity	kWh	946	
Botanicals	kg	22.5

**Table 2:** Mass balance of main inputs and outputs for the production of one batch of gin from wheat, based on Arbikie commercial production.

Input/output	Dry matter kg	Starch kg	Protein kg	Volume L
Whole grain	2703	1865	341	
Pot-ale (DDGS)	1092		341	10547
Alcohol				1159
Gin				1886

**Table 3:** Mass balance of main inputs and outputs for the production of one batch of gin from peas, based on Arbikie pilot trials.

Input/output	Dry matter kg	Starch kg	Protein kg	Volume L
Whole grain	4558	2338	1089	
Hulls	1777		347	
Grist	2782	1419	743	
Pot-ale (DDGS)	1363		743	10547
Alcohol				1159
Gin				1886

**Table 4:** Mass balance of main inputs and outputs for the production of one batch of gin from peas, based on equivalent starch input to fermentation.

Input/output	Dry matter	Carbohydrates	Starch	Protein	Volume L
	kg				
Whole grain	5905	3319	3030	1412	
Hulls	2301	1373		655	
Grist	3604	1946	1838	757	
Pot-ale	1766	108		757	10547
Alcohol					1159
Gin					1886

**Table 5:** Activity data used to parameterise LCA of pea and wheat cultivation.

Cultivation phase	Pea	Wheat	Unit
Inputs			
Fertiliser N – Ammonium nitrate^a^^,^^b^	0	119	kg/ha
Fertiliser N – Urea^a^^,^^b^	0	44	kg/ha
Fertiliser P_2_O_5_^a^^,^^b^	40	40	kg/ha
Fertiliser K_2_O^a^^,^^b^	20	60	kg/ha
Lime^c^	250	500	kg/ha
Agrochemicals (Active ingredient)^a^^,^^d^	1.4	4.6	kg/ha
Seeds^a^^,^^d^	125	204	kg/ha
Diesel^e^	52.5	63.5	L/ha
Outputs			
Grains (@85% dry matter)^a^^,^^f^	4810	7430	kg/ha
Straw (@80% dry matter)^a^	NA	2993	Kg/ha

a Arbike Estate Farm Manager, pers. Comm.

b UK Fertiliser Manual[5].

c UK Fertiliser use survey[6].

d James Hutton Institute Farm Manager, pers. Comm.

e Calculated from activity data multiplied by energy use coefficients from Dalgaard et al[7].

f PGRO pea agronomy guide[8].

**Table 6:** Crude protein and metabolizable energy contents of cattle feeds.

Parameter	Pea hulls	Wheat DDGS	Pea DDGS	Soybean meal	Barley grain
Dry matter (DM), % fresh matter	90	90	90	88	87
Crude protein, kg kg^−1^ DM	0.19	0.35	0.55	0.52	0.11
Metabolizable energy (MJ kg^−1^ DM)	8.8	12.5	12.5	11.95	12.4

**Table 7:** Quantities of soybean meal and barley grain substituted (negative values) by pea hulls and wheat- and pea-based DDGS, per batch of gin.

Co-product	Total crude protein (kg)	Total metabolizable energy (MJ)	Substituted soybean meal (kg)	Balancing barley grain (kg)
Pea hulls (1777 kg DM)	330	15635	−547	−842
Wheat DDGS (1092 kg DM)	341	13650	−628	−569
Pea DDGS (1363 kg DM)	743	17038	−1696	+300

**Table 8:** Life cycle impact assessment methods employed in this study.

Impact category	Indicator	Unit	Recommended default LCIA method	Source of CFs	Robustness	Selected method in OpenLCA
Climate change	Radiative forcing as Global Warming Potential (GWP100)	kg CO2 eq	Baseline model of 100 years of the IPCC (based on IPCC 2013)	EC-JRC, 201721	I	IPCC 2013
Ozone depletion	Ozone Depletion Potential (ODP)	kg CFC-11 eq	Steady-state ODPs as in (WMO 1999)	EC-JRC, 2017	I	ILCD+
Human toxicity, cancer*	Comparative Toxic Unit for humans (CTUh)	CTUh	USEtox model (Rosenbaum et al., 2008)	EC-JRC, 2017	III/interim	ILCD+
Human toxicity, non-cancer*	Comparative Toxic Unit for humans (CTUh)	CTUh	USEtox model (Rosenbaum	EC-JRC,	III/interim	ILCD+
Ionising radiation, human health	Human exposure efficiency relative to U235	kBq U235 eq	Human health effect model as developed by Dreicer et al., 1995 (Frischknecht et al., 2000)	EC-JRC, 2017	II	ILCD+
Photochemical ozone formation, human health	Tropospheric ozone concentration increase	kg NMVOC eq	LOTOS-EUROS model (Van Zelm et al., 2008) as implemented in ReCiPe 2008	EC-JRC, 2017	II	ILCD+
Acidification	Accumulated Exceedance (AE)	mol H+ eq	Accumulated Exceedance (Seppälä et al., 2006, Posch et al., 2008)	EC-JRC, 2017	II	ILCD+
Eutrophication, terrestrial	Accumulated Exceedance (AE)	mol N eq	Accumulated Exceedance (Seppälä et al., 2006, Posch et al., 2008)	EC-JRC, 2017	II	ILCD+
Eutrophication, freshwater	Fraction of nutrients reaching freshwater end compartment (P)	kg P eq	EUTREND model (Struijs et al., 2009) as implemented in ReCiPe	EC-JRC, 2017	II	ILCD+
Eutrophication, marine	Fraction of nutrients reaching marine end compartment (N)	kg N eq	EUTREND model (Struijs et al., 2009) as implemented in ReCiPe	EC-JRC, 2017	II	ILCD+
Ecotoxicity, freshwater*	Comparative Toxic Unit for ecosystems (CTUe)	CTUe	USEtox model, (Rosenbaum et al., 2008)	EC-JRC, 2017	III/interim	ILCD+
Resource use, minerals and metals	Abiotic resource depletion (ADP ultimate reserves)	kg Sb eq	CML 2002 (Guinée et al., 2002) and van Oers et al., 2002.		III	CML IA Baseline
Resource use, fossils	Abiotic resource depletion – fossil fuels (ADP-fossil)	MJ	CML 2002 (Guinée et al., 2002) and van Oers et al., 2002	EC-JRC, 2017	III	CML IA Baseline
Land occupation	Cropping land occupation (LO)	m^2^.yr			II	NA

Key data outputs are summarised in Tables within the associated MS Excel file, including: (i) life cycle inventory data (Table SI 9 for wheat gin and Tables SI 10 and SI 11 for wheat gin produced at different alcohol yields); (ii) life cycle assessment results broken down into 11 contributory processes and the four life cycle assessment permutations evaluated in Lienhardt et al. [1], in Tables SI 12–SI 15.

## 2 Experimental design, materials, and methods

### 2.1 Input and output mass balance

Data from Arbikie on input quantities to the distillation process (Table 1), and from Feedipedia [2] on pea and wheat grain composition, were used to derive mass balances of macro nutrients for the production of one batch of gin (1886 L) from wheat (Table 2) and peas (Table 3). The alcohol production from fermentation (1159 L) is within 2% of the specific alcohol yield per kg of wheat grain reported by Ref. [3], and within 7% of the stoichiometric yield of alcohol from the carbohydrate content of pea grist [4].

To reflect some uncertainty in alcohol yields for pea flour at the commercial scale, we also undertook an LCA of pea gin based on an equivalent carbohydrate input from pea flour (1946 kg) as from wheat grist (Table 4). This represents a 30% higher input of peas compared with data provided by Arbikie, and may be regarded as a worst case estimate of alcohol production efficiency from peas.

### 2.2 Cultivation and field emissions

Table 5 displays major inputs and outputs expressed per hectare for wheat and pea cultivation, based on a combination of specific activity data from the Arbikie Estate (where wheat is grown for the distillery) and national statistics.

Soil emissions and nutrient leaching factors following the application of synthetic and organic fertilizers were primarily taken from relevant inventory reports [9], [10], [11]. Nitrogen losses from pot ale spreading were calculated based on the MANNER-NPK tool [12] which integrates equations derived from decades of empirical observations across the UK on emissions, leaching and fertiliser replacement value for different organic nutrient additions [12]. Ammonia emissions and N leaching are related to factors including total N, NH_4_ and dry matter contents of organic amendments, application method, soil type and moisture status during application, cropping sequence, and prevailing meteorological conditions during and after application (as specified by users and inferred from background meteorological data related to the post code). The soil hydrological balance is also important for calculating N leaching. We ran the MANNER-NPK tool for pot ale application by trailing hose in spring and autumn, under good spreading conditions (calm weather, moist soils, no rain immediately after application), on a medium textured soil prior to a spring cereal crop.

Credits for avoided fertiliser application comprised avoided manufacture taken from the Ecoinvent database [13] and avoided field emissions post-application based on emission factors of 0.017 NH_3_–N^11^, 0.1 NO_3_–N [14] and 0.01 for P following N- and P-fertiliser application [15]. Unless otherwise stated, nitrogen, phosphorus and potassium fertilisers were assumed to be in the forms of ammonium nitrate, triple superphosphate and potassium chloride fertilisers.

### 2.3 Avoided animal feed

Pea hulls and pot ale (following conversion to dried distillers grains with solubles, DDGS) may be used as cattle feed, substituting a mix of protein- and energy-feeds. Based on the same approach as Leinonen et al. [16], we assumed that soybean meal and barley were the main feeds substituted. We applied linear optimisation run in MS Excel solver to calculate the amount of soybean meal and barley grain substituted by pea hulls, wheat-based DDGS and pea-based DDGS in order to deliver exactly the same amount of crude protein and metabolizable energy. Crude protein and metabolizable energy content values for the different feed stuffs (Table 6) were taken from Feedipedia [2]. The protein content of pea-derived DDGS was calculated based on the protein mass balance in Table 7. The mass balance of animal feed substitution following optimisation is displayed in Table 7. In the case of pea-based DDGS, substitution of soybean meal leaves a deficit of metabolizable energy, which is satisfied by feeding additional barley grain (a burden that offsets some of the feed substitution credit calculated in the expanded boundary LCA).

### 2.4 Impact assessment

Life cycle impact assessment was undertaken across 14 environmental impact categories (Table 8). Thirteen of these are from the suite of impact assessment methods recommended for the European Product Environmental Footprint (PEF) harmonisation initiative [17]. We took all these methods that were available in OpenLCA v.1.7.4. This resulted in the exclusion of the following PEF-recommended impact categories: Particulate Matter, Water Resource Depletion and Land Use & Soil Quality. Owing to the important land use implications of wheat substitution with peas in gin production, we represented Land Occupation with a simple metric of m^2^.yr of cropland required [18], using inventory data reported in Ecoinvent v3.5 [13] (Table SI8).

## 3 Results

Tables SI 9–SI 11 summarise life cycle inventory inputs and outputs underpinning the LCA results across 14 impact categories (Table 8) and 11 key contributory process categories. Tables SI 12–SI 15 provide results for four LCA permutations: (i) attributional LCA of gin, with pot-ale treated as a waste product; (ii) attributional LCA of gin, with allocation across gin and pot-ale as an animal feed co-product; (iii) expanded boundary LCA with pot-ale used as a bio-fertiliser substituting synthetic fertiliser; (iv) expanded-boundary LCA, with pot-ale used as an animal feed substituting soybean and barley.
